# 1781. Antibiotic Prescription Trends of Patients with COVID-19: Analysis of National Health Insurance Data in Republic of Korea

**DOI:** 10.1093/ofid/ofac492.1411

**Published:** 2022-12-15

**Authors:** Yunsang Choi, Minsun Kang, Dong Hoon Shin, Jongtak Jung, Seong Jin Choi, Nak-Hyun Kim, Song Mi Moon, Kyoung-Ho Song, Eu Suk Kim, Jaehun Jung, Hong Bin Kim

**Affiliations:** Seoul National University Bundang Hospital, Seoungnam-si, Kyonggi-do, Republic of Korea; Gil Medical Centre, Gachon University College of Medicine, Incheon, Inch'on-jikhalsi, Republic of Korea; Seoul National University College of Medicine, Seoul, Seoul-t'ukpyolsi, Republic of Korea; Soonchunhyang University Seoul Hospital, Seoul, Seoul-t'ukpyolsi, Republic of Korea; Seoul National University College of Medicine, Seoul, Seoul-t'ukpyolsi, Republic of Korea; Seoul National University Bundang Hospital, Seoungnam-si, Kyonggi-do, Republic of Korea; Seoul National University College of Medicine, Seoul, Seoul-t'ukpyolsi, Republic of Korea; Seoul National University College of Medicine, Seoul, Seoul-t'ukpyolsi, Republic of Korea; Seoul National University College of Medicine, Seoul, Seoul-t'ukpyolsi, Republic of Korea; Gil Medical Centre, Gachon University College of Medicine, Incheon, Inch'on-jikhalsi, Republic of Korea; Seoul National University College of Medicine, Seoul, Seoul-t'ukpyolsi, Republic of Korea

## Abstract

**Background:**

Although COVID-19 is a viral infection, it is known that antibiotics are often prescribed due to concerns about combined bacterial infection. Therefore, we aimed to analyze how many patients with COVID-19 received the antibiotic prescription as well as what kinds of factors contributed to it using the National Health Insurance database.

**Methods:**

We retrospectively reviewed claims data for adults 19 years of age and older hospitalized for COVID-19 from December 1, 2019 to December 31, 2020. According to severity classification of the National Institutes of Health guidelines, we calculated not only the proportion of patients receiving antibiotics but also days of treatment per 1000 patient days. In addition, we investigated the factors contributing to antibiotic use by linear regression analysis.

**Results:**

Of the 55,228 patients, 47% were male, 55% were older than 50 years of age, and most patients (89%) had no underlying diseases. The majority (84%, 46,576) were classified as having mild to moderate illness, with 11% (6,168) and 5% (2,484) having severe and critical, respectively. Antibiotics were prescribed in a total of 27% (15,081). While 74% of patients with severe illness and 88% of those with critical illness received antibiotic treatment, even 18% of mild to moderate cases were prescribed antibiotics. Fluoroquinolones were the most commonly prescribed antibiotics (8,348), followed by third generation cephalosporins (5,729) and beta-lactam/beta-lactamase inhibitors (3,822) as shown in Figure 1. Older age, severity of disease and underlying medical conditions contributed to overall prescription rates as well as days of antibiotic use significantly (Table 1).

**Figure:**
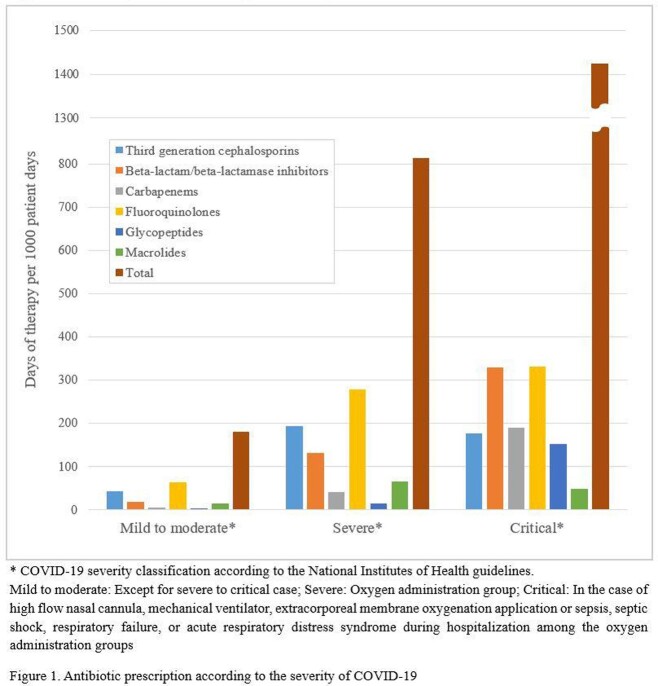

**Figure:**
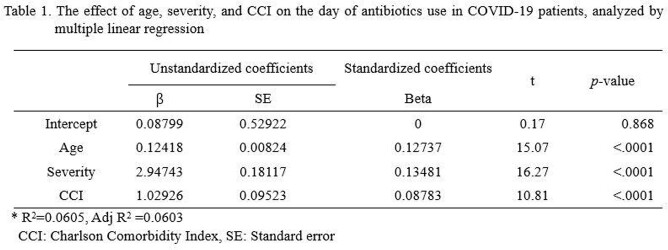

**Conclusion:**

Although most of COVID-19 patients had mild to moderate illness, more than a quarter were prescribed antibiotics. Judicious use of broad-spectrum antibiotics is necessary for COVID-19 patients, considering the severity of disease and the risk of bacterial co-infection.

**Disclosures:**

**All Authors**: No reported disclosures.

